# The Nipah virus threat in India: Epidemiological trends, risk drivers, and lessons for pandemic preparedness

**DOI:** 10.1371/journal.ppat.1014226

**Published:** 2026-05-20

**Authors:** Apoorva, Sunit K. Singh

**Affiliations:** 1 Molecular Biology Unit, Faculty of Medicine, Institute of Medical Sciences, Banaras Hindu University, Varanasi, Uttar Pradesh, India; 2 Dr. B.R. Ambedkar Center for Biomedical Research (ACBR), University of Delhi, Delhi, India; 3 Delhi School of Public Health (DSPH), University of Delhi, Delhi, India; Fred Hutchinson Cancer Center, UNITED STATES OF AMERICA

## Abstract

Nipah virus (NiV), a WHO-priority zoonotic pathogen, poses a recurrent public health threat in India. Since its initial detection in 2001, India has encountered multiple NiV spillover events, including repeated outbreaks in Kerala and recent cases reported in West Bengal in 2026. Although the pig-amplified epidemic in Malaysia (1998–1999) and the seasonal outbreaks associated with NiV-contaminated date palm sap consumption in Bangladesh are extensively documented, India represents a unique and relatively understudied epidemiological setting. NiV outbreaks in India are primarily linked to the NiV-Bangladesh clade and are marked by direct spillover transmission from bats to humans, elevated case-fatality rates, clustered emergence, and effective household/nosocomial transmission without an intermediate amplifying host. Spillover risk is influenced by region-specific ecological and socio-economic factors, including contamination of peri-domestic fruit, proximity of bat roosts to human settlements, deforestation, and urban expansion. To date, no treatment or vaccine is available for humans or animals. This review synthesizes evidence from India spanning 2001 to 2026. It integrates aspects of epidemiology, bat reservoir ecology, viral genomics, clinical manifestations, surveillance experiences, and health system responses, while contrasting India’s transmission dynamics with those observed in Malaysia and Bangladesh. Furthermore, we explore India’s current preparedness and response strategies, highlighting the critical shift from reactive outbreak control to a proactive, integrated “One Health” framework. The strategy to strengthen integrated surveillance, contact tracing, isolation, and quarantine of suspected and confirmed cases, advance India-developed vaccines and therapeutics, and address the root ecological drivers of zoonotic emergence is essential for national and global health security.

## 1. Introduction

Nipah virus (NiV), an emerging zoonotic paramyxovirus, is the etiological agent of Nipah virus disease (NVD) [1]. It was first identified during a large outbreak of encephalitis and respiratory illness in Malaysia in 1998 [2,3]. It primarily affects pigs and humans. It is named after Sungai Nipah, a Malaysian village from which the virus was first isolated. The outbreak resulted in significant socio-economic disruption, including mass culling of over one million pigs and 109 human fatalities [2]. Since then, the epidemiological distribution of NiV outbreaks has notably expanded, with recurrent cases reported in Bangladesh and India [4]. The highest incidence is observed in Bangladesh, followed by Malaysia, India, the Philippines, and Singapore [5]. The Centers for Disease Control (CDC) classifies NiV as a Category C bioterrorism agent. NiV has also been categorized as a WHO priority (biosafety risk group 4) pathogen due to its high (40% to 75%) case-fatality rate (CFR), ability to cause severe neurological and respiratory diseases, documented human-to-human transmissions, and lack of licensed vaccines or therapeutics [6,7].

In India, the NiV outbreak was initially identified in West Bengal in 2001 (Siliguri) and 2007 (Nadia) [8,9]. After a decade of inactivity, the virus reemerged in Kerala, a southern Indian state, in 2018 [10,11]. Since then, multiple localized outbreaks have been reported in the years 2019, 2021, 2023, 2024, and 2025 [12,13]. Recently, two new cases of NiV have been reported in West Bengal (January 2026) [14,15]. The repeated occurrence of NiV in India highlights its increasing endemic potential, characterized by heightened human-bat interactions, ecological disturbances, and climate change [16–18]. Indian outbreaks are marked by direct spillover from fruit bats (*Pteropus medius*) and notable transmission within familial and healthcare environments [8,19]. Unlike in Malaysian outbreaks, where pigs served as significant intermediate hosts, direct spillover from bats to humans is the primary mode of transmission in India [2]. The interplay of India’s substantial population, varied ecosystems, and changing human-animal interactions necessitates a comprehensive understanding of NiV epidemiology, transmission dynamics, and pathogenesis. This review summarizes the existing scientific understanding of the NiV in the Indian context, identifies existing knowledge gaps, and proposes strategic One Health approaches for preparedness and response in an era characterized by climate change and ecological flux. The review was conducted via a systematic literature search across PubMed, Web of Science, Scopus, and Google Scholar for studies published from 1998 until January 2026, utilizing combinations of the terms “Nipah virus,” “India,” “pathogenesis,” “Kerala,” “West Bengal”, “diagnosis,” “transmission,” “therapy,” “vaccine,” and “outbreak.” Emphasis was placed on peer-reviewed original studies, recent systematic reviews, outbreak reports, and guidance documents from WHO, NCDC, ICMR, and relevant government health agencies, all pertaining to the Indian context.

## 2. Epidemiology: India’s two decades of Nipah virus outbreaks

The epidemiology of NiV in India shows how a zoonotic pathogen establishes a precarious presence across various geographical and socio-ecological contexts (Table 1). Unlike the large, single epidemic in Malaysia, India has faced multiple, smaller, localized outbreaks (Table 2) [2,9,11,12,20]. Outbreaks in India have predominantly occurred in two specific geographical regions: West Bengal in the east and Kerala in the south [9,11–13,20,21]. Why does this unique spatial pattern exist? Understanding why NiV outbreaks appear so geographically confined, clustering repeatedly in these two states (West Bengal and Kerala), is crucial to understanding the epidemiology of NiV in India. The answer is probably not attributable to a single factor, but rather to a combination of ecological and demographic conditions. Historically, the first Indian outbreaks occurred in 2001 in Siliguri and in 2007 in Nadia, West Bengal, along the India–Bangladesh border [8,9]. The exact origin of zoonotic spillover, although believed to involve fruit bats, was not clearly established. However, the region exhibits ecological similarities with Bangladesh, where NiV epidemics are common [22]. The Siliguri and Nadia districts of West Bengal are characterized by high population density, the presence of *Pteropus medius* fruit bats, and socio-cultural practices, including the consumption of raw date palm sap. The outbreaks in West Bengal were marked by acute encephalitis in adults [9]. The spread of NiV infection in healthcare environments indicates a significant role of nosocomial transmission [8,9]. However, since 2018, Kerala has emerged as the primary NiV hotspot [11,20]. The recurrent outbreaks documented in Kozhikode, Malappuram, Ernakulam, and Palakkad districts of Kerala from 2018 to 2025 mark a clear geographical shift in India’s NiV landscape [10–13,20]. The ecological landscape of Kerala, situated within the Western Ghats biodiversity hotspot, sustains dense bat populations and fosters interactions between wildlife and human settlements via fragmented forests, commercial fruit orchards, and backyard fruit trees [10,23–25]. The conditions promote recurrent zoonotic spillover, primarily linked to bat-contaminated fruits rather than date palm sap consumption. Nevertheless, rapid case detection, aggressive contact tracing, and stringent infection-control practices have significantly reduced secondary transmission, transforming potential large outbreaks into single-case events in 2019, 2021, 2024, and 2025 [17,26]. Intriguingly, seasonality differentiates the two regions and highlights ecological consistencies. Outbreaks in West Bengal generally occurred during the cooler pre-monsoon season (January–March), which coincides with the seasonal risk period in Bangladesh [18]. In contrast, outbreaks in Kerala primarily occur during the monsoon and post-monsoon months (May–September), which align with bat breeding cycles and fruit-harvesting seasons. Specific fruits (such as mangoes, sapota, and dates) attract fruit bats. Human activities, such as date palm sap, facilitate contact with bat-contaminated products. Nonetheless, the practice of tapping date palm sap collection is less prevalent in Kerala than in Bangladesh. Furthermore, seasonal variations, such as changes in temperature and precipitation, can affect bat physiology, reproductive patterns, and foraging behaviors, potentially leading to heightened viral shedding and increased human exposure to contaminated food sources [27]. Periods of food scarcity in bats and habitat destruction may drive them to forage near human settlements or depend on cultivated fruits, thereby elevating the risk of spillover [22,27].

**Table 1:** Epidemiology of Nipah virus cases in India.

Year	Location	Cases reported	Mode of Detection	CFR (%)	Secondary Transmission	Clinical manifestations	Key features	References
2001	Siliguri, West Bengal	66	Serology (IgM ELISA)	68	Nosocomial (45% HCWs); 23 family clusters	Fever, encephalitis, respiratory distress	First Indian outbreak; hospital amplification	[9]
2007	Nadia, West Bengal	5	RT-PCR confirmation	100	Family cluster	Acute encephalitis, seizures	Rapid containment; small outbreak	[8]
2018	Kozhikode, Kerala	23	RT-PCR (throat swab)	91	14 hospital contacts; 2 family clusters	Encephalitis (17/19), ARDS (7/19)	Student index; m102.4 used	[11]
2019	Ernakulam, Kerala	1	RT-PCR (saliva)	0	None	Fever, myalgia, recovered	Isolated case; survived supportive care	[25]
2021	Kozhikode, Kerala	1	RT-PCR (CSF)	100	None	Encephalitis, coma	12-year-old boy; rapid detection	[55]
2023	Kozhikode, Kerala	6	Truenat PoC (swabs)	33	Limited family contacts	Encephalitis (4), pneumonia (2)	Remdesivir protocol acquired; lowest CFR	[17]
2024	Malappuram, Kerala	2	RT-PCR (blood)	100	None	Acute respiratory failure	Spillover in fruiting season	[141]
2025	Malappuram/Palakkad, Kerala	4	Truenat + RT-PCR	50	Under investigation	Encephalitis, seizures	First Palakkad cases; geographic expansion	[142]
2026	Barasat, West Bengal (ongoing)	2 (ongoing)	RT-PCR (NIV mobile)	–	Healthcare workers	Fever, respiratory	West Bengal resurgence	[15,143]

**Table 2:** Comparative epidemiology, pathogenesis, and clinical manifestations of Nipah virus outbreaks.

Characteristic/Feature	India	Bangladesh	Malaysia
Period	2001–present	2001–present	1998–1999
Case-fatality rate	High (33–100%)	Very high (40–90%)	Low (~40%)
Epidemiological pattern	Recurrent, sporadic outbreaks	Recurrent seasonal outbreaks (Annual Nipah belt)	Single large epidemic
Seasonality	Post-monsoon (regional)	Winter (date palm sap-harvesting season)	No clear seasonality
Dominant viral lineage	NiV-B clade	NiV-B clade	NiV-M clade
Bat reservoir host	*Pteropus medius*	*Pteropus* spp.	*Pteropus* spp.
Intermediate amplifying host	Absent	Absent	Pigs
Primary route of spillover	Direct bat-to-human transmission (contaminated fruit, peri-domestic exposure)	Bat-to-human (contaminated date palm sap consumption)	Bat-to-pig-to-human
Socio-ecological factors	Deforestation, land-use change, and bat-human proximity	Cultural practices (raw date palm sap consumption)	Intensive pig farming
Human-to-human transmission	Frequent, including nosocomial	Common household transmission	Minimal
Nosocomial amplification	Prominent	Occasional	Rare
Incubation period	1–14 days	1–14 days	4–18 days
Onset of illness	More frequently abrupt onset	More frequently, abrupt onset	Often gradual, with prominent prodromal symptoms
Disease progression	Rapid	Rapid	Slow
Respiratory symptoms	More prominent; commonly includes cough, dyspnea, and atypical pneumonia	More prominent; commonly includes cough, dyspnea, and atypical pneumonia	Less frequent; may include cough and sore throat
Gastrointestinal symptoms	Vomiting and diarrhea are less common	Vomiting and diarrhea are less common	Vomiting and diarrhea may occur
Neurological symptoms	Encephalitis, seizures, and other neurological signs (myoclonus less frequent)	Encephalitis, seizures, and other neurological signs (myoclonus less frequent)	Encephalitis, seizures, myoclonus (more frequent), and other neurological signs
Clinical presentation	Acute encephalitis, severe pneumonia & ARDS	Encephalitis, respiratory illness	Predominantly encephalitis
Long-term sequelae	Reported persistent neurological deficits, seizures, and personality changes	Reported persistent neurological deficits, seizures, and personality changes	Rare
Prevention and control	Rapid containment via One Health	Community-based prevention measures	Mass culling of pigs

**Fig 1:**
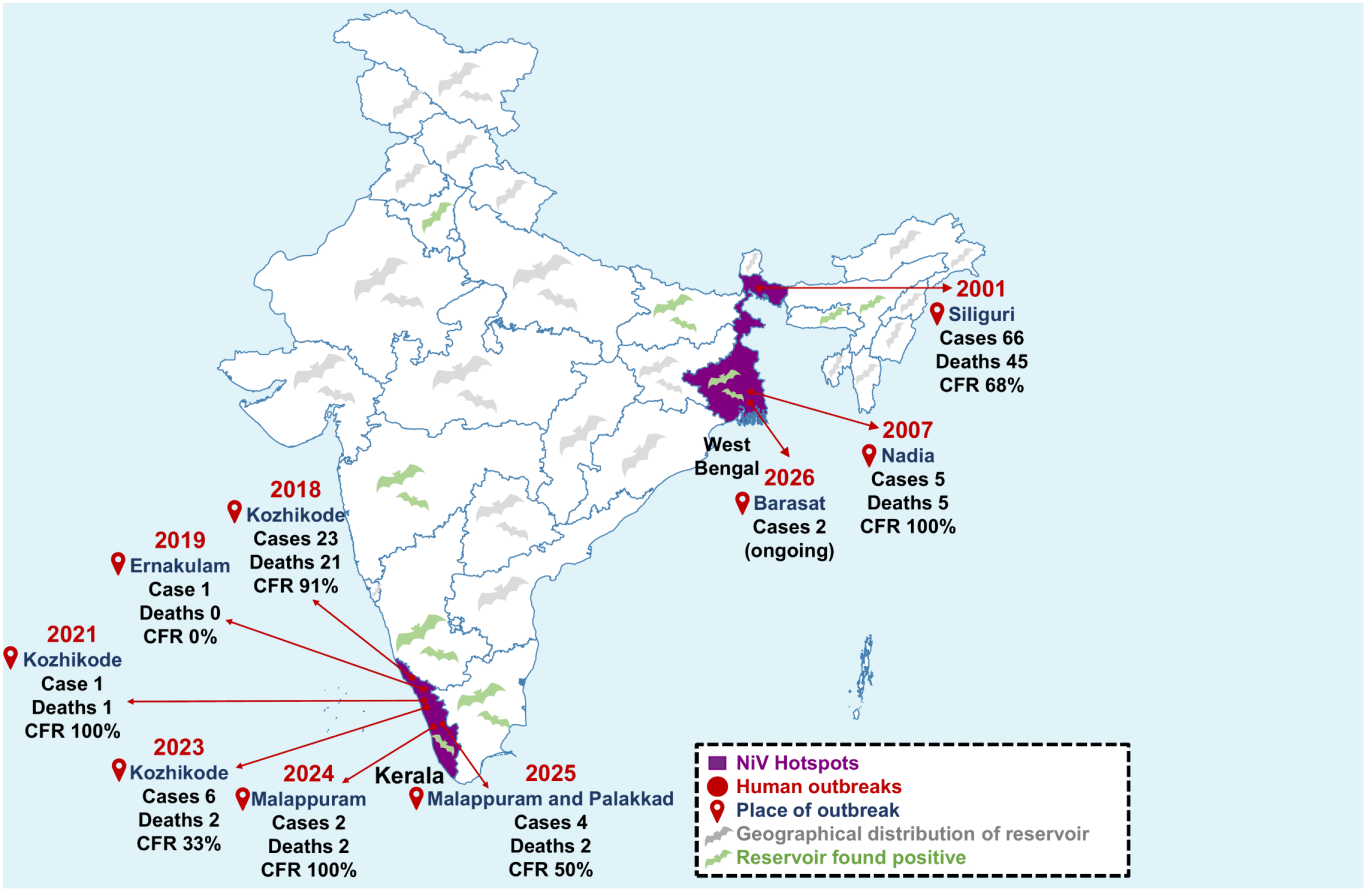
Epidemiological distribution of Nipah virus outbreaks and reservoirs in India (2001–2026). The map depicts NiV epicenters in India, highlighting the eastern (West Bengal) and southern (Kerala) as high-risk zones for zoonotic spillover due to high bat density and frequent bat-human interactions. Red circles indicate locations of confirmed human outbreaks. Labels denote the year of emergence, the count of laboratory-confirmed cases, and the Case-Fatality Rate (CFR). The gray bats delineate the established geographical distribution of the primary natural reservoir. The green bats reflect regions where NiV RNA was molecularly discovered in bat tissues or anti-NiV IgG antibodies were found, indicating extensive circulation outside epidemic zones. Base map data sourced from geoBoundaries (https://www.geoboundaries.org/) licensed under CC BY 4.0. Map processed and visualized using Mapshaper (https://mapshaper.org/). The authors added outbreak data overlays, labels, and annotations.

In India, NiV outbreaks have reported CFRs of 45%–100%, predominantly in Kerala and West Bengal, indicating the virus’s high pathogenicity and the lack of specific antiviral treatments (Fig 1). The high CFR is attributable to the virulence of the NiV-Bangladesh (NiV-B) clade. The 2001 Siliguri outbreak in West Bengal documented 66 probable cases and 45 fatalities, resulting in a CFR of 68%. The outbreak was exacerbated nosocomially following an undetected index case at Siliguri District Hospital, which infected 11 patients, leading to additional transmission to 25 employees and 8 visitors [9]. The 2007 Nadia outbreak involved 5 cases, resulting in 100% CFR. It was associated with the consumption of date palm-derived alcohol in the index case, with subsequent transmission affecting one healthcare worker [8]. The 2018 outbreak in Kozhikode–Malappuram, Kerala, documented 18 confirmed cases and 17 fatalities, resulting in a CFR of 94%. The outbreak predominantly impacted individuals of economically productive age, without gender bias, and included at least one nosocomial infection within healthcare settings [11,17,20]. Recent outbreak data in Kerala show variable CFRs: 0% in 2019 (Ernakulam; 1 case), 100% in 2021 (Kozhikode; 1 case involving a 12-year-old boy post-rambutan consumption), 33% in 2023 (Kozhikode; 6 cases), 100% in 2024 (Malappuram; 2 cases), and 50% in 2025 (Malappuram–Palakkad; 4 cases from May to July, including an 18-year-old woman and a 14-year-old boy) [12,13,21]. The high CFR in India is attributable to the virulence of the (NiV-B) lineage, which dominates Indian outbreaks, delayed identification due to nonspecific early symptoms, and inadequate access to sophisticated critical care in some places.

Demographically, NiV infection impacts all age groups in India; however, exposure risk influences case distribution. Adult males are slightly overrepresented among confirmed cases, possibly because of their occupational roles in agriculture, fruit-harvesting, and caregiving, which increase their proximity to bat habitats and contaminated products. The 2025 Kerala outbreak involved cases of an 18-year-old woman and a 14-year-old boy from Malappuram, underscoring the participation of both adolescents and adults. Healthcare workers and family caregivers are at significant risk during human-to-human transmission in nosocomial and household settings, as evidenced by studies in Siliguri (2001) and Kozhikode (2018), respectively [9,11,28]. The notable familial clustering and nosocomial amplification highlight the necessity for effective infection-control measures in both clinical and home environments [11]. Socio-economic factors increase risks for populations residing near forests or involved in subsistence agriculture, as evidenced by the ongoing hotspots in Kerala, despite the lack of date palm sap practices [27,29]. While this evidence strongly suggests Kerala and West Bengal as major “hotspots,” it is crucial to evaluate whether these two regions are the only “hotspots,” or whether they are merely the locations with adequate surveillance systems and diagnostic capabilities for virus detection. This distinction is epidemiologically significant, as it is plausible that NiV spillovers remain undetected in other regions, masked by insufficient diagnostic resources. Serological and ecological studies have detected NiV in *Pteropus medius* populations outside of the two outbreak states, including Assam, Maharashtra, and other areas, indicating a wider enzootic circulation of NiV in India. Due to the severe nature and high CFR of clinically recognized NiV disease, widespread unnoticed symptomatic transmission is unlikely; however, sporadic cases manifesting as acute encephalitis syndrome or severe respiratory illness may be misidentified in the absence of laboratory testing. Most importantly, asymptomatic transmission and asymptomatic infection are distinct considerations. While asymptomatic transmission is unlikely given the disease’s severity, asymptomatic or mildly symptomatic infections may still occur and remain undetected, creating a critical gap in outbreak detection. Although these infections are unlikely to drive sustained transmission, they cannot be disregarded and necessitate focused sero-surveillance in high-risk groups to capture the true infection burden and quantify the frequency of spillover events that would otherwise go unrecognized. Enhancing syndromic surveillance and broadening molecular diagnostics beyond established hotspots may more accurately delineate the actual prevalence and geographic distribution of Nipah virus infection in India.

## 3. Genomic structure and replication

NiV is an enveloped paramyxovirus characterized by a nonsegmented, negative-sense RNA genome (18.2 kb in length) [30]. The NiV genome encodes six proteins: nucleocapsid (N), phosphoprotein (P), matrix (M), fusion glycoprotein (F), attachment glycoprotein (G), and long polymerase (L) [31]. The viral RNA associates with N, P, and L proteins to form a ribonucleoprotein complex. Meanwhile, F and G proteins, which are integrated into the lipid membrane, enable virus attachment and entry into host cells through ephrin-B2 and ephrin-B3 receptors, which are prevalent in endothelial cells, neurons, and various other tissues [32,33]. The P gene generates nonstructural proteins (V, W, and C) via mRNA editing and alternative open reading frames (ORF), which are essential for the virus’s pathogenicity [34]. The V protein primarily inhibits cytoplasmic antiviral sensing pathways by interacting with RNA helicases like MDA5 and obstructing downstream interferon signaling through the sequestration of STAT1 and STAT2 [35]. The W protein, produced through RNA editing, is localized in the nucleus, where it inhibits host antiviral responses by disrupting IRF3-mediated activation of the IFN-β promoter and altering nuclear innate immune signaling [36,37]. The C protein, derived from an alternative open reading frame (ORF), plays a role in virulence by influencing viral replication efficiency, modulating host inflammatory responses, and improving pathogenic fitness in vivo [38]. Studies using reverse genetics in hamster models demonstrate that deleting proteins V or C significantly reduces disease severity, whereas deleting protein W does not. This suggests that proteins V and C are critical factors in the pathogenesis of Nipah virus, extending beyond their established roles as interferon antagonists [38]. The L protein comprises RNA-dependent RNA polymerase (RdRp) and Polymerase and Ribonucleotide Transferase (PRNTase) domains, which engage with the P protein’s zinc-binding modules to establish the viral polymerase complex [39,40].

**Fig 2:**
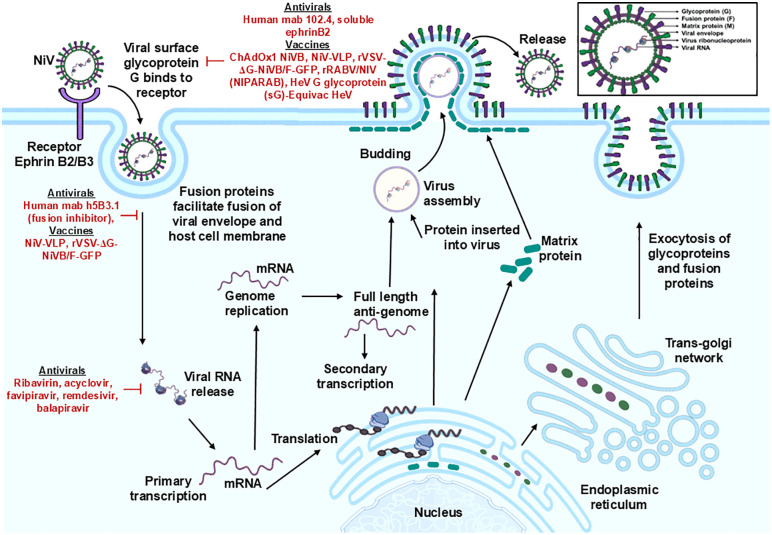
The replication cycle of the Nipah virus. The schematic outlines the sequential stages of the NiV life cycle, such as attachment to ephrin-B2/B3 receptors through the G glycoprotein, F protein-mediated membrane fusion, release of the viral RNA, replication and transcription, translation of viral proteins (N, P, M, F, G, and L), assembly, encapsidation, and budding of progeny virions from the host cell membrane. Vaccine candidates and antiviral agents that target crucial stages of the viral life cycle are highlighted in red. Icons were used from publicly available resources including NIH/NIAID BioArt Source (https://bioart.niaid.nih.gov/), Servier Medical Art (https://smart.servier.com/; CC BY 4.0), BioIcons (https://bioicons.com/;CC0), and the Reactome Icon Library (https://reactome.org/icon-lib; CC BY 4.0). Final figure design, layout, and annotations were created by the authors.

NiV infections are marked by the formation of endothelial syncytia, inflammation, and hemorrhagic manifestations, caused by viral cytopathic effects and dysregulated host cytokine responses. The replication cycle begins with the binding of the G protein to ephrin-B2 and ephrin-B3 receptors on the host cell membrane, facilitating the F protein-mediated fusion of the viral envelope with the host cell membrane. The fusion process releases the viral nucleocapsid into the cytoplasm, where viral RNA is transcribed and replicated by the viral RdRp. The positive-sense mRNA is subsequently capped and polyadenylated in the cytoplasm, mimicking host mRNA to enhance translation by the host’s ribosomes. The newly synthesized RNA genomes are encapsulated by the N protein, forming new ribonucleoprotein complexes. These complexes assemble at the host cell membrane, orchestrated by the M protein, to generate new virions. Virions bud off, acquiring a lipid envelope that contains G and F glycoproteins, which facilitate the infection of new cells. The cycle of infection, reproduction, and transmission promotes swift viral dissemination and significant morbidity (Fig 2) [41].

Mutations in surface proteins G and F, such as G273S and Y228H, can alter the virus’s structural dynamics, thereby improving its binding affinity for the Ephrin B2 receptor and augmenting its infectivity [42]. Molecular dynamics simulations indicate that these mutations enhance the virus’s adaptability to diverse hosts and environments by introducing flexibility and conformational alterations in the protein complexes, thereby complicating therapeutic interventions [43]. Furthermore, Guillaume et al. identified a NiV-G protein residue, E533, which plays a crucial role in receptor-binding and exhibits structural and functional similarities to the measles virus attachment hemagglutinin residue, R533 [44]. Through 3D modeling of the NiV-G protein, the researchers identified six additional protein residues (W504, E505, N557, Q530, T531, and A532) that appear to play significant roles in facilitating viral fusion and binding to ephrin-B2, a functional receptor for NiV present on epithelial cells and neurons [33,44,45]. Aguilar et al. demonstrated that the NiV-F protein undergoes glycosylation at several sites, which diminishes its fusion efficiency relative to mutated F proteins, unlike the role of N-glycans in other paramyxoviruses. The authors found that NiV N-glycans may contribute to the protein’s resistance to neutralizing antibodies [46]. Recent deep mutational scanning (DMS) studies have significantly enhanced the residue-centric framework by facilitating systematic, high-throughput mapping of the functional consequences of numerous mutations across the NiV receptor-binding protein (RBP/G) and F protein [47,48]. The analyses indicate that mutations at the RBP dimer interface, such as R258 and F266, significantly impair cell entry, highlighting their importance in preserving structural integrity and facilitating the conformational coupling necessary for F activation. Moreover, mutations at critical receptor-interacting regions, including sites N557 and Y581, as well as positions within the 580–590 loop and site 492, have been demonstrated to differentially influence ephrin-B2 and ephrin-B3 binding [47]. This refines previous low-throughput observations and elucidates the mechanisms that govern receptor specificity and tropism. The F protein demonstrates significantly greater functional constraint, as most substitutions hinder fusion competence. However, certain mutations in regions such as the heptad repeat A (HRA) can be tolerated and may aid prefusion stabilization [48]. Additionally, the mapping of antibody escape landscapes indicates that mutations in functionally constrained regions tend to be detrimental, whereas more permissive regions of the RBP can tolerate substitutions that facilitate immune evasion without significantly compromising viral fitness [47]. Comparison with Indian Nipah virus isolates, specifically the Kerala 2018 and West Bengal 2007 strains, reveals significant conservation of critical residues, with no indications of escape-associated substitutions at essential functional sites [8,10]. Clade-specific polymorphisms are observed outside of major constrained or antigenic regions, indicating the maintenance of receptor usage and antigenicity despite genetic divergence. These findings suggest that isolated mutations can influence receptor affinity or fusion efficiency; however, the mutational landscape of NiV G and F is significantly constrained, allowing for minimal evolutionary variation at functionally critical sites. The NiV-G and F proteins are essential for host cell binding, fusion, and viral budding. Nonetheless, their role in this process is limited relative to that of the viral matrix protein M, which appears to be essential for viral organization and budding [30].

**Fig 3:**
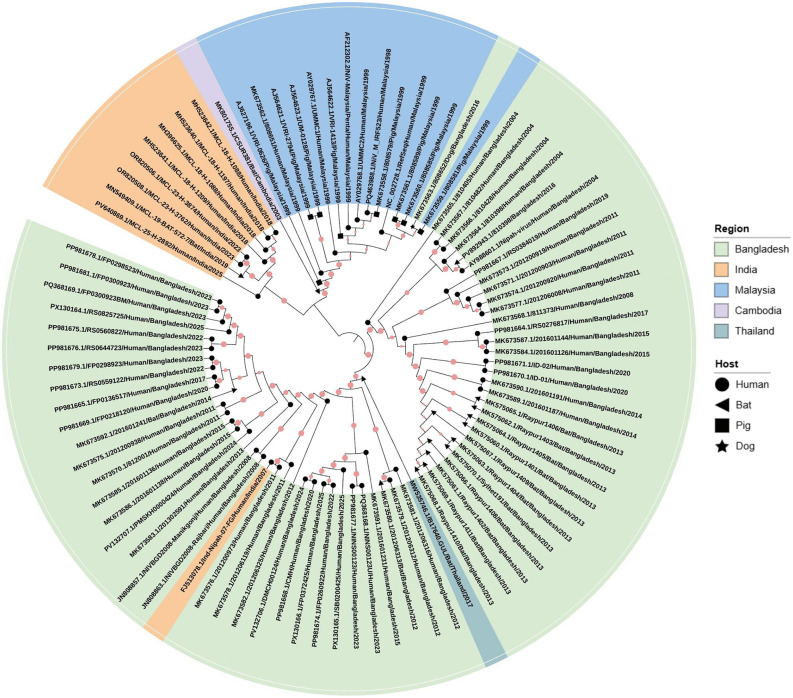
Molecular phylogenetic analysis of Nipah virus complete genome sequences. The evolutionary history of NiV was inferred using the Maximum-Likelihood (ML) method in MEGA (Molecular Evolutionary Genetics Analysis; https://www.megasoftware.net/) using 89 complete genome sequences obtained from GenBank (National Center for Biotechnology Information; https://www.ncbi.nlm.nih.gov/genbank/). Sequence alignment and phylogenetic reconstruction utilized the General Time Reversible (GTR) nucleotide substitution model, involving a discrete Gamma distribution (+G) across 5 categories to address rate heterogeneity among sites (shape parameter = 3.7546) and a proportion of evolutionarily invariant sites (+I = 59.29%). The final dataset included 18,252 nucleotide positions post-alignment trimming. The initial heuristic search tree was chosen based on the superior log-likelihood score of Neighbor-Joining (NJ) compared to Maximum Parsimony (MP) starting trees. Statistical robustness of the inferred topology was assessed using 501 bootstrap replicates; branches that appeared in fewer than 50% of the replicates were collapsed. Internal node support values are indicated by red circles, with circle size (0.96–1) proportional to bootstrap support. The tree was visualized and annotated by the Interactive Tree of Life (iTOL) platform (https://itol.embl.de/). The outer color strips denote the geographic origin of isolates. Symbols at the branch tips indicate the host species. Labels are formatted as accession number, host, country, and year.

Phylogenetic analyses show that Indian NiV isolates cluster exclusively within the NiV-B clade, unlike the NiV-Malaysia (NiV-M) clade, which includes Malaysian, Cambodian, and some Thai sequences [3,49,50]. To examine the evolutionary relationships among NiV isolates, we analyzed 89 publicly available complete genome sequences from GenBank using a maximum-likelihood phylogenetic approach (Fig 3). G gene nucleotide similarity for NiV-B strain, including Indian isolates, is 98–100%, whereas inter-clade similarity is 92.2–93%. N gene patterns have 99.1–100% inter- and 93.6–94.6% intra-clade similarity [49]. Indian NiV-B genomes have 91.8% similarity with NiV-M and six extra nucleotides in the F gene 5′ nontranslated region, as well as V gene variation required for interferon suppression and immune evasion [51,52]. Genetic stability during acute infection is supported by low intra-host heterogeneity (4/18,246 nucleotides between throat and CSF isolates [8]. In contrast to Malaysia’s 1998–1999 outbreak, characterized by the rapid spread of a single pig-adapted variant with nearly identical human and pig sequences, India’s recurrent bat-to-human spillovers resemble the pattern observed in Bangladesh. This situation promotes increased inter-strain heterogeneity, indicative of multiple zoonotic introductions [53,54]. A robust 729-nucleotide genotyping approach for Bangladesh 2008–2010 sequences establishes a reliable framework for classifying Indian NiV-B isolates, facilitating origin tracking and outbreak linkage with strong bootstrap support [51]. Sub-clades in Kerala, exhibiting over 99% similarity to those from West Bengal in 2007, suggest regional persistence instead of external reintroduction, in contrast to the singular amplification event observed in Malaysia [55,56]. The distinctions between pig-centric transmission in Malaysia and human-centric transmission in India and Bangladesh correlate with genetic divergence and higher CFRs in India compared to Malaysia [50]. This highlights the need for targeted surveillance and specific countermeasures.

## 4. Pathogenesis and clinical manifestation

NiV exhibits a range of disease severity, from asymptomatic cases to rapidly progressing disease. Manifestations typically occur within two weeks of NiV exposure in humans (>90% of cases), although the incubation period may extend up to 2 months (Tables 2 and 3) [1]. The duration may vary depending on factors such as the mode of transmission and patient demographics [30]. Rare instances of delayed-onset or relapsing encephalitis have been documented months to years post-initial infection, especially after the Malaysian outbreak, indicating potential viral persistence in the CNS. In contrast, outbreaks in India have predominantly exhibited acute presentations with high CFR and limited evidence of relapse, probably due to the size of the outbreaks and genotype-specific variations [57,58]. Evidence from Indian NiV outbreaks suggests that the clinicopathological progression is consistent with previous descriptions from Malaysia and Bangladesh, characterized by acute febrile illness advancing to encephalitis, respiratory compromise, and multisystem endothelial injury [9,11,59]. Notably, brainstem dysfunction, including myoclonus, abnormal ocular reflexes, autonomic instability, and impaired consciousness, was a hallmark of the Malaysian outbreak and correlated with a poor prognosis, highlighting the significance of brainstem involvement in severe NiV neuropathogenesis [60]. Autopsy and clinical pathology reports have identified vasculitis changes and neuronal involvement, which align with established henipavirus pathology [61,62]. Reports from the 2018 Kerala outbreak further documented significant pulmonary involvement, including pneumonia and ARDS in severe cases, alongside radiological and clinical evidence of encephalitic disease [11,28]. Several Indian case series have reported cardiac involvement, including myocarditis; however, the frequency and lineage-specific significance of this observation remain unclear [63]. Robust comparisons between Indian NiV-B and Malaysian NiV-M outbreaks are limited by small case numbers, heterogeneous clinical reporting, and the absence of systematic postmortem studies.

**Table 3:** Comparative epidemiology, pathogenesis, and clinical manifestations of Nipah virus outbreaks in India.

Characteristic/Feature	West Bengal	Kerala
Year	2001, 2007, 2026	2018–2025
Districts	Siliguri, Nadia and North 24 Parganas	Kozhikode, Malappuram, and Palakkad
Outbreaks	3	9
Outbreak size	Small (5–9 cases/outbreak)	Variable (1–23 cases/outbreak)
Case-fatality rate	High (68%–100%)	Very high (33%–100%)
Epidemiological pattern	Sporadic outbreaks	Recurrent seasonal outbreaks
Seasonality	Not established	Post-monsoon (Sep-Oct)
Dominant viral lineage	NiV-B clade	NiV-B clade
Intermediate amplifying host	Absent	Absent
Primary route of spillover	Bat-to-human (contaminated date palm sap consumption, peri-domestic exposure)	Bat-to-human (contaminated fruit, peri-domestic exposure, bat proximity)
Socio-ecological factors	Urbanization, Bangladesh proximity	Bat habitat encroachment
Human-to-human transmission	Frequent household transmission	Prominent household transmission
Nosocomial amplification	Frequent	Prominent
Incubation period	1–14 days	1–14 days
Onset of illness	More frequently abrupt onset	More frequently, abrupt onset
Disease progression	Rapid	Rapid
Respiratory symptoms	Common	Prominent
Gastrointestinal symptoms	Less common	Less common
Neurological symptoms	Encephalitis, seizures, and other neurological signs	Encephalitis, seizures, and other neurological signs
Clinical presentation	Predominantly encephalitic	Acute encephalitis, severe pneumonia & ARDS
Long-term sequelae	Not well-documented	Fatigue, neurological deficits
Prevention and control	ICMR rapid response, BSL-3 deployment	State preparedness, contact tracing, and rapid containment via One Health

**Fig 4:**
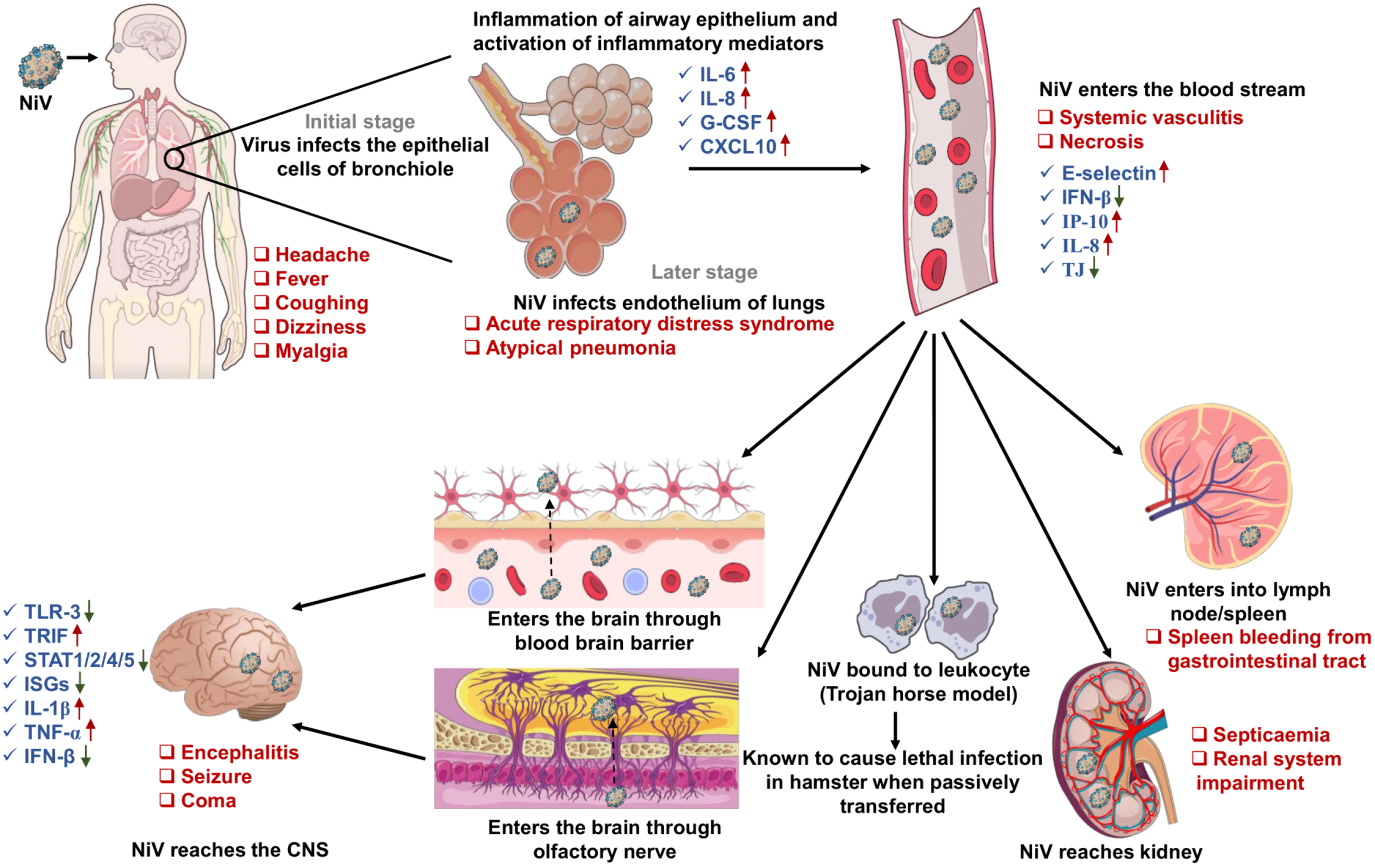
Pathophysiology of Nipah virus infection. This diagram depicts the pathogenesis of NiV after respiratory entry. Following the initial infection of bronchial epithelial cells, the virus disseminates to lung endothelial cells, eliciting a significant inflammatory response. Viral dissemination into the bloodstream facilitates systemic spread, potentially through a “Trojan horse” mechanism involving infected leukocytes and the participation of lymphoid organs and kidneys. Neuroinvasion occurs via hematogenous dissemination across the blood-brain barrier or via retrograde transport along the olfactory nerve, leading to encephalitis, seizures, coma, and brainstem dysfunction. Pulmonary infections can lead to acute respiratory distress syndrome (ARDS) and atypical pneumonia, with systemic endothelial injury and inflammation contributing to multi-organ dysfunction. Icons were used from publicly available resources including NIH/NIAID BioArt Source (https://bioart.niaid.nih.gov/), Servier Medical Art (https://smart.servier.com/; CC BY 4.0), and the Reactome Icon Library (https://reactome.org/icon-lib; CC BY 4.0). Final figure design, layout, and annotations were created by the authors.

NiV predominantly enters the human body through the oral-nasal route, likely via exposure to infected bat secretions or contaminated food (Fig 4) [55]. After mucosal entry, initial replication is believed to occur in the respiratory tract, with tropism for bronchial epithelial cells and type II pneumocytes, as evidenced by experimental infection models and corroborated by histopathological analyses of related henipavirus systems [64]. The initial replication phase is characterized by early systemic symptoms, including fever, headache, myalgia, vomiting, and malaise [65]. The virus then infiltrates lung endothelial cells, triggering a robust inflammatory response characterized by cytokine production (IL-6, IL-8, G-CSF, and CXCL10), often resulting in pathology similar to acute respiratory distress syndrome (ARDS) [66]. The substantial respiratory involvement is a characteristic feature of the NiV-B strain, which distinguishes Indian outbreaks from those caused by the Malaysian strain (NiV-M), which primarily resulted in encephalitis with relatively minimal pulmonary disease [3]. Primarily, interferon (IFN) signaling represents the first line of defense against viral infection, rapidly establishing an antiviral state via type I IFN (IFN-α/β) production. Pattern recognition receptors (PRRs), such as TLR3, TLR7, TLR9, RIG-I, and MDA5, identify viral RNA and rapidly activate IFN signaling, which is crucial for early antiviral immunity. NiV has evolved strategies to evade the host’s immune defense. NiV P gene products (V, C, W proteins) effectively inhibit IFN responses by blocking the IRF3 phosphorylation, STAT1/2 nuclear translocation, and IFN-β production through cytoplasmic retention (P/V proteins) or nuclear sequestration (W protein), creating a dual cytoplasmic-nuclear block [35,37,67,68]. Experimental mutagenesis has identified specific residues within the shared STAT1-binding motif, notably Y116, as essential factors influencing interferon antagonism and virulence [67]. The virus also interacts with the E3-ubiquitin ligase TRIM6 to suppress IκB kinase epsilon (IKKe), thereby inhibiting type I interferon (IFN-I) signaling, promoting viral dissemination, and evading antiviral responses [69]. Furthermore, NiV P gene–encoded proteins, particularly P, V, and W, sequester STAT1 and STAT2 within inclusion bodies, inhibiting their activation and nuclear translocation, thereby preventing the activation of antiviral genes [70]. In contrast, research on differentiated porcine airway epithelial cells has reported elevated expression of type I/II IFN-related genes, suggesting partial activation of the IFN response that may inhibit viral replication [71]. Moreover, NiV inhibits IFN response genes, including Ifna7 and Iigp1, illustrating its capacity to evade the host’s innate immune system [72]. The W protein suppresses proinflammatory cytokine production and enhances virulence by promoting the nuclear retention of the cellular scaffold protein 14-3-3, thereby diminishing phosphorylation and nuclear entry of the NF-κB p65 subunit [36]. Subsequent to these early responses, neutrophils are recruited to the infection site. These cells release reactive oxygen species (ROS), antimicrobial peptides, and neutrophil extracellular traps (NETs), which can immobilize and neutralize viral particles [41]. While these mechanisms successfully immobilize viral particles, excessive activation can exacerbate tissue damage and promote immune dysregulation, potentially aiding viral persistence [73]. Cellular immunity, particularly through CD4+ and CD8+ T cells, is essential for viral clearance. Most of the evidence comes from animal models. In nonhuman primate and porcine models, CD8⁺ cytotoxic T lymphocytes are activated during the acute phase of infection, as indicated by markers such as Ki67, granzyme B, and PD-1, underscoring their role as effector cells that target and eliminate infected cells. CD4+ T cells facilitate the immune response by releasing cytokines [74]. However, direct evidence in humans remains limited; survivors from Bangladesh have shown persistent NiV-specific CD4⁺ and CD8⁺ T-cell responses, including IFN-γ and IL-2 reactivity to F and G peptides, detectable decades after infection [75]. In addition, B cells produce antibodies in response to viral antigens in humoral immunity. The generation of specific antibodies may take up to 2 weeks; however, memory B cells enable a more rapid response during subsequent exposures. Research on NiV survivors has revealed detectable IgG and IgM antibodies, indicating a protective function during both the acute and convalescent phases [68]. Recent studies using NiVΔF replicon particles in hamsters demonstrated broad IgG and IgA responses with Fc-mediated effector functions, suggesting that mechanisms such as ADCC and phagocytosis may also contribute substantially to protection [76]. Furthermore, studies on reservoir hosts have demonstrated that bats’ unique immune regulation and broad repertoire of naive immunoglobulins contribute to prolonged viral persistence and delayed antibody responses [77,78]. In vivo studies in NiV-infected pigs show that significant levels of neutralizing antibodies develop within 2 weeks; however, viral RNA persists, indicating delayed virus eradication [41]. B cell depletion in African green monkeys (AGMs) accelerates disease progression, with survival observed exclusively in those with IgM and IgG responses [79].

Systemic viral dissemination occurs via free virions and leukocyte-associated “Trojan horse” transport, facilitating immune evasion and multi-organ spread [80,81]. This process enables infection of endothelial cells in the lungs, spleen, and kidneys, leading to multi-organ mechanisms: hematogenous dissemination across a compromised blood-brain barrier (BBB), and direct neuroinvasion through the olfactory nerve. The CNS neuroinvasion pathway is still poorly understood in human infection and has primarily been inferred from animal model studies [82,83]. The strong affinity for the CNS is primarily due to its ability to disrupt the BBB, allowing direct infection of neurons and microglial cells. This elicits a strong inflammatory response, marked by the secretion of cytokines and chemokines (IL-6, IL-8, IL1β, IFNβ, TNFα, TRIF, and TLR-3), that could contribute to encephalitis, seizures, confusion, and, in severe instances, coma within 24–48 hours of infection [40]. Magnetic resonance imaging (MRI) demonstrates multifocal hyperintense lesions affecting the cerebral cortex, pons, temporal lobes, and white matter, with diffusion-weighted imaging facilitating differentiation from other viral encephalitis [84,85]. NiV infection is characterized by relapsing or late-onset encephalitis, which can manifest weeks to years after apparent recovery or subsequent to asymptomatic infection. Over 20 cases have been documented, including one occurring more than a decade after infection, marked by extensive confluent cortical MRI lesions and pathological evidence of ongoing CNS vasculopathy [59,84]. Individuals who survive NiV infection may experience prolonged neurological consequences, such as gait disturbances, neuropsychiatric symptoms, cognitive impairment, and chronic fatigue that can persist for years after the initial infection [86].

## 5. Reservoir ecology of Nipah virus in India

Fruit bats of the genus *Pteropus* have been identified as the primary natural reservoirs of NiV, sustaining asymptomatic infections and intermittently shedding virus-contaminated urine, saliva, and feces into the environment [25,87]. In contrast to humans, NiV does not induce overt disease in bats, facilitating persistent circulation and longevity within bat metapopulations [88]. *Pteropus medius*, formerly known as *P. giganteus*, dominates NiV detections across the Indian outbreak and border sites, although reports of secondary species such as *Rousettus leschenaultia* in Kerala and West Bengal, and *Pipistrellus sp*. in Maharashtra have been reported [89]. NiV has been identified in bats across various regions of India, including northern (Haryana), eastern (West Bengal, Bihar), northeastern (Assam, Meghalaya), western (Maharashtra), and southern region (Kerala, Karnataka, Tamil Nadu, and Puducherry) [9,10,19,23–25,56,87,89,90]. This distribution suggests a broader prevalence of the virus across India than indicated by the limited number of documented human outbreaks [56,87,89,90]. The initial documentation of NiV exposure in Indian bats occurred in Haryana during 2003–2004, as evidenced by serological testing [9]. The first molecular identification of NiV RNA in Indian bats occurred in 2009–2010 in Myanaguri, West Bengal [87]. Viral RNA was subsequently detected in Cooch Behar (West Bengal) and Dhubri (Assam) in 2015, Kozhikode (Kerala) in 2018, Ernakulam and Idukki (Kerala) in 2019, and Wayanad (Kerala) in 2023 [23,25,90]. Furthermore, the known NiV infectious zone was expanded westward with the discovery of NiV RNA in bats from Bihar in 2022–2023 [56]. Yadav et al. (2018) and Balasubramanian et al. (2024) found that viral RNA is more frequently detected in visceral organs such as the spleen, kidney, and liver, whereas rectal and throat swabs are often negative, suggesting that viral shedding is episodic rather than continuous [56,90]. Serological surveys conducted throughout India indicate variable NiV seroprevalence in *P. medius* populations, ranging from 9% to 65%, influenced by factors such as geographic location, year of sampling, and ecological conditions [23,25,55,56]. In Kerala, seroprevalence also shows slight temporal variation (9% in February, 24% in July, and 28% in September), suggesting weak seasonality rather than stringent climatic control [23,25]. Conversely, eastern and northeastern states, including West Bengal, Assam, Bihar, and Meghalaya, often exhibit elevated seropositivity rates (23–65%), especially in samples gathered in May [56]. The identification of anti-NiV IgG antibodies in juvenile bats in India indicates recent enzootic transmission, as the rapid decline of maternal antibodies results in a susceptible cohort that maintains viral circulation [25,56]. High seroprevalence may transiently reduce viral transmission; however, immunity reduces over time, facilitating periodic epizootics in bat colonies [88].

## 6. Transmission dynamics of Nipah virus in India

**Fig 5:**
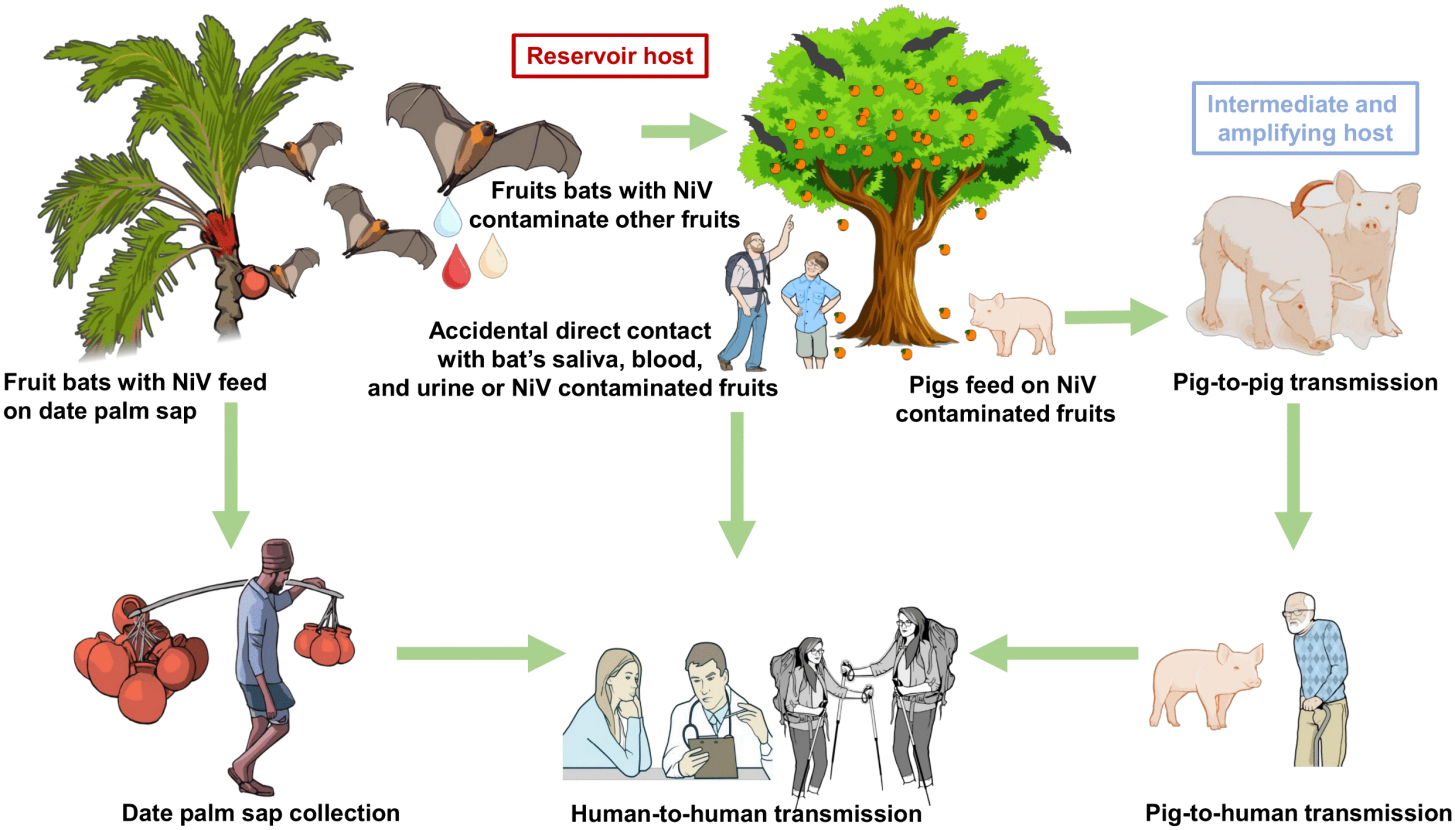
Transmission dynamics of Nipah virus. The schematic presents an overview of NiV transmission from reservoir hosts to humans either via consumption of contaminated raw date palm sap/fruits or through contact with intermediate amplification hosts. Icons were used from publicly available resources including NIH/NIAID BioArt Source (https://bioart.niaid.nih.gov/) and Openclipart (https://openclipart.org/). Final figure design, layout, and annotations were created by the authors.

NiV transmission in India is characterized by recurrent zoonotic spillover from native fruit bat reservoirs (*P. medius*), accompanied by frequent human-to-human transmission, especially within familial and healthcare settings (Fig 5) [10,19]. Since Indian outbreaks are primarily attributed to the NiV-B clade, which significantly differs from the NiV-M strain, it is characterized by high virulence and enhanced transmissibility [29,54,91]. In contrast to the Malaysian epidemic, characterized by pig-mediated amplification and restricted secondary transmission, Indian outbreaks occur via direct bat-to-human transmission without an intermediate host. Genomic analyses demonstrate over 99% homology between bat- and human-derived strains, thereby confirming reservoir-origin viral spillover [90]. Conversely, Bangladesh demonstrates pronounced winter seasonality influenced by the consumption of raw date palm sap, with recurrent outbreaks occurring in the “Nipah belt”, where high-density bat roosts [88,92,93]. In addition, due to deforestation, land-use change, and urban expansion, *P. medius* often forages near human settlements, facilitating viral spillover through contaminated fruits, surfaces, and environmental substrates [88,94].

Human-to-human transmission is a key epidemiological characteristic of NiV in India, primarily occurring through close contact with infected individuals, exposure to contaminated bodily fluids, respiratory droplets, and fomites [28,95,96]. This transmission pattern is prominent in both nosocomial and household clusters. The 2001 Siliguri outbreak in West Bengal demonstrated significant hospital-based transmission, with around 75% of cases involving healthcare workers and visitors, attributed to patient transfers and public healthcare facilities [9]. The virus’s high transmission potential became apparent during the 2018 Kozhikode outbreak in Kerala, where a single index case led to 15 secondary infections, including among healthcare professionals, with a median incubation period of 9 days [11]. The highly mobile population of Kerala increases the risk of transmission Nipah virus infection. In addition, NiV’s basic reproduction number (R₀) in India is estimated to range from 0.3 to 2.0, and it occasionally exceeds epidemic thresholds in high-contact healthcare settings [97,98]. Although the average R₀ typically remains below 1, sporadic transmission chains may arise if immediate isolation is delayed. In contrast, in Bangladesh, secondary transmission is prevalent but typically confined to family clusters. In India, especially in Kerala, robust syndromic surveillance and rapid response capabilities under the Integrated Disease Surveillance Program (IDSP) have facilitated the early detection of isolated spillovers, evidenced by the lack of secondary transmission during outbreaks from 2019 to 2025 [26,99]. Case exposures remain unconfirmed but epidemiologically linked to peri-domestic bat roosts (e.g., Kerala’s 2018 Kozhikode index near fruit trees; West Bengal 2026 Barasat nurses’ regional travel); severity scales with viral dose, and environmental persistence amplifies fomite risks. This direct bat-human interface and the absence of intermediate amplification underpin India’s sporadic high CFR (68–94%) spillovers. NiV also exhibits substantial environmental stability, remaining viable for up to 7 days at 22 °C in simulated date palm sap and for at least 3 days in fruit juices, and retaining infectivity for short durations at elevated temperatures [65,100]. Collectively, these ecological, behavioral, and infrastructural elements establish a unique Indian transmission profile characterized by frequent direct spillover, efficient human-to-human transmission, elevated mortality, and recurring emergence (Table 4). This highlights the necessity for a One Health-oriented surveillance strategy that integrates bat ecology, land-use monitoring, and early human case identification to avert future outbreaks.

**Table 4:** Bat reservoir ecology and spillover risk of Nipah virus in India.

Characteristics	Description/ Evidence	Spillover risk	References
Primary reservoir species	*Pteropus medius*	Endemic viral circulation	[9,25]
Geographic distribution	Commonly found throughout peninsular and eastern India, particularly in Kerala and West Bengal	Facilitates recurrent and spatially distinct spillover effects	[27]
Roosting ecology	Established, enduring colonies in urban, peri-urban, and agricultural environments	Continuous vicinity to human populations	[27]
Feeding behavior	Frugivorous	Fruit contamination with saliva, feces, and urine	[11]
Evidence of viral circulation	NiV RNA and anti-NiV antibodies detected in Kerala bats	Shows continuous circulation irrespective of outbreaks	[23,25]
Seasonal viral shedding	Increased southern India viral detection post-monsoon	Aligns with the temporal clustering of cases	[23]
Human-bat interfaces	Proximity of bat roosts to residential areas, orchards, and community water sources	Facilitates direct transmission from bats to humans	[27]
Role of intermediate hosts	No persistent intermediate amplifying hosts in India	Direct spillover enhances unpredictability	[11]
Anthropogenic drivers	Increased agriculture, urbanization, and deforestation	Alters bat habitats and elevates contact rates	[27]
Climate-related influences	Heat stress, altered fruiting, and habitat alterations	Alters bat migration and viral excretion	[27]
Spillover pathway	Exposure to contaminated fruit or environments directly	Distinct from pig-mediated and sap-associated routes	[92,93,131]
Knowledge gaps	Insufficient longitudinal surveillance of bats; inadequate mapping of roost dynamics	Reduces early-warning and predictive modeling	[27]

## 7. Climate change and anthropogenic activities as key determinants of Nipah virus spillover in India

Climate change and anthropogenic land-use alteration synergistically disrupt *P. medius* ecology, intensifying stress-induced viral shedding and increased human-bat interactions, catalyzing NiV spillover in India [94,101]. Indian Council of Medical Research (ICMR)–National Institute of Virology (NIV) surveillance showed continuous viral circulation and >99% genetic similarity between bat and human isolates during the 2018 Kerala epidemic [10], indicating recurring zoonotic spillover rather than episodic reintroduction. Habitat fragmentation resulting from deforestation, agricultural intensification, and peri-urban expansion in the Western Ghats and eastern India has led to the displacement of *P. medius* into densely populated areas, thereby reducing the ecological distance between reservoir and host [17,23,55]. The decline of native fruiting trees increases bat foraging in domestic gardens and orchards, thereby heightening the potential for direct transmission. Climate-induced changes in temperature and precipitation further reshape bat migration ecology and seasonal behavior. Projected warming trends are expected to expand the latitudinal and elevational distribution of *P. medius*, while erratic monsoon patterns disrupt natural fruiting phenology, necessitating bats to engage in short-distance migratory movements (15–45 km) into human-dominated habitats during times of resource scarcity [88]. Indian outbreaks are concentrated during the hot, humid months of May to September, aligning with peak fruiting, breeding, and juvenile recruitment periods. The periods are characterized by heightened bat movement, foraging in peri-urban areas, and long-distance roost shifts exceeding 100 km [102]. Notably, four out of six outbreaks in Kerala occurred during El Niño years, which aligns with the El Niño-Southern Oscillation (ENSO)-related drought that preceded Malaysia’s outbreak in 1998–1999 [3,63]. Evidence from both experimental and field studies favors the stress-shedding hypothesis in which nutritional stress, heatwaves, and immunomodulation enhance viral excretion in bats, consequently increasing the risk of spillover [27,103,104]. These results imply that India’s NiV transmission dynamics are increasingly shaped by climate-modulated ecological processes rather than fixed cultural practices alone. In addition, Kerala’s epidemics highlighted maladaptive social reactions that may increase risk. This practice could facilitate broader viral dissemination by dispersing infected colonies and simultaneously disrupting ecosystem services (pollination and seed dispersal). These events have also resulted in significant economic losses, including the collapse of fruit and cash crop markets, despite a lack of evidence for foodborne transmission via exported products [23]. These dynamics also suggest that India may be moving toward a spillover regime similar to that of Bangladesh, where ecological degradation supports ongoing emergence [22]. Although the exact effects of climate change on NiV transmission are not well understood, accumulating evidence suggests that the interplay of climatic and anthropogenic factors and the lack of intermediary amplifier hosts, like pigs (in Malaysia), reduces the ecological distance between bats and humans, making India’s transmission dynamics vulnerable to climate-induced behavioral shifts and rapid landscape transformation.

## 8. Diagnostics and surveillance

Diagnosis of NiV infection in India remains challenging as initial symptoms (fever, headache, and respiratory distress) are nonspecific and seroconversion is often delayed, with IgM antibodies detectable only after 7–15 days [30]. Logistical obstacles in remote regions further complicate the diagnosis [10]. Real-time RT-PCR that targets conserved N/P genes from CSF, blood, throat swabs, or urine is the gold-standard for acute detection (sensitivity >98%, ICMR-NIV, Pune) (Table 5). Serological IgM/IgG ELISA (recombinant N/G protein) supports convalescent and epidemiological diagnosis, achieving 100% positivity by day 12. Virus isolation and neutralization tests are confined to a biosafety level (BSL)-4 facility, currently limited to the ICMR-NIV Pune; however, initial processing can be done at BSL-3 [1]. The use of a mobile BSL-3 laboratory during the 2023 Kerala epidemic enabled on-site molecular diagnosis, underscoring the need for decentralized capabilities [40]. The introduction of Truenat Point-of-Care micro-PCR in 2019 at tertiary centers enables rapid field-based confirmation within 1 hour during outbreaks [105]. It has demonstrated high sensitivity (97%; 95% CI 90–100) and specificity (100%; 95% CI 98–100) [106]. National guidelines define suspected cases and 21-day contact tracing. However, due to fragmented genomic surveillance, delays in transporting samples from remote areas, the absence of operational BSL-3 facilities in hotspots, and episodic bat RNA detection despite seroprevalence, owing to shedding intermittency, these limit diagnostic capability [23]. In contrast to Bangladesh’s nationally integrated Nipah surveillance system, India’s surveillance remains dispersed, with organized syndromic and contact-based monitoring primarily limited to Kerala. Advanced NiV detection methods such as microfluidic RT-PCR–lateral flow hybrids, one-pot RPA-CRISPR/Cas13a and RAA-CRISPR/Cas12a with a fluorescence quantification system capable of electricity-free, field-stable operation, with limits of detection as low as 10 copies/μL, are not available in India [106–109]. These techniques outperform traditional RT-PCR and ELISA in terms of speed and portability, enabling multiplexed strain differentiation and real-time ecological surveillance.

**Table 5:** Diagnostic and surveillance for Nipah virus in India.

Diagnostic modality	Specimen type	Sensitivity	Application	Availability in Indian context	Limitations	References
qRT-PCR (N, M, P genes)	Nasal/throat swab, CSF, blood, urine	High; detects ~10–10³ copies	Acute diagnosis	Primary frontline test; available at ICMR-NIV Pune and few VRDLs	Cold-chain dependency; limited access in remote districts	[30,55]
Truenat PoC micro-PCR	Nasal/throat swab, blood	High	Rapid on-field diagnosis	Deployed since 2019 in Kerala (14+ sites); WB mobile 2026	Limited national coverage	[105]
Nested RT-PCR	Nasal/throat swab, blood, CSF, urine	High	Confirmatory	Used at reference laboratories	Contamination risk; labor-intensive	[144]
SYBR Green qRT-PCR	Homogenized tissues	Moderate-high	Quantification	Research settings	Lower specificity	[145]
PRNT	Serum	Very high	Gold-standard	Only at BSL-4 labs	Resource-intensive	[146]
Competitive ELISA (cELISA)	Serum	High	Screening	Research purpose	Requires PRNT confirmation	[147]
ELISA (IgM/IgG)	Serum, CSF	High	Retrospective diagnosis, serosurveys	Used in outbreak investigations	Not useful for early infection	[55]
Virus isolation	Swabs, tissues	High	Confirmation and research	Only at BSL-4 labs	Extreme biosafety requirements	[1]
Bat surveillance (PCR + serology)	Urine, saliva, serum	Low RNA detection	Spillover risk assessment	Episodic sampling	Infrequent shedding	[23]

## 9. Progress and challenges associated with vaccine and therapeutics development against the Nipah virus

Currently, no antiviral or vaccine has been authorized for the prevention or treatment of NiV infection in humans, and clinical management primarily consists of supportive care, including intensive monitoring, respiratory and neurological support, seizure management, and prevention of secondary complications [110]. However, significant advancements have been achieved in the preclinical development of antivirals, monoclonal antibodies (mAbs), and vaccine platforms, propelled by NiV’s elevated CFR and its prioritization under the WHO R&D Blueprint (Table 6). Therapeutic research has concentrated on broad-spectrum nucleoside analogues and host-directed strategies. Remdesivir, an adenosine analogue, provided full protection in AGMs if administered early; however, prolonged therapy resulted in decreased efficiency and persistent neuropathology [111]. Favipiravir, a purine analogue that targets RdRp, provided 100% protection in Syrian hamsters infected with NiV-M [112]. However, more recent studies in hamsters using both NiV-M and NiV-B strains revealed variable, and often partial, efficacy [113]. Balapiravir (R1479), a cytidine analogue, demonstrates *in vitro* inhibition of NiV and Hendra virus (HeV); however, its application is constrained by toxicity and inadequate bioavailability in other viral infections [39]. Ribavirin, a guanosine analogue, has produced contradictory outcomes, with moderate mortality reductions in Malaysian cohorts but inconsistent findings in Indian case series and trials; its significance in encephalitic illness is still debated [85]. PolyI‑polyC12U, an interferon inducer, demonstrates complete *in vitro* inhibition and significantly enhances survival in hamsters. In contrast, chloroquine, while inhibiting henipavirus entry via cathepsin L *in vitro*, does not confer protection *in vivo* [114]. Case series from Singapore and India indicate the empirical use of acyclovir but lack clear efficacy [16]. Consequently, these agents have limited translational value in comparison to candidates demonstrating reproducible *in vivo* protection. In Kerala outbreaks, the implementation of compassionate protocols that included early remdesivir and favipiravir prophylaxis for high-risk contacts was associated with a lower CFR than in previous epidemics. However, the reliability of this effect is constrained by small sample sizes and potential selection bias [17]. The mAbs provide highly specific therapeutic options for pathogens. The m102.4, a human phage-display-derived neutralizing antibody which targets the HeV/NiV G glycoprotein receptor-binding domain, has shown complete protection in ferrets and AGMs against NiV-M and HeV. It exhibits a narrower therapeutic window for NiV-B and has been found to be safe, nonimmunogenic, and pharmacokinetically linear in a phase 1 trial involving healthy adults [115,116]. Recent in vivo studies have shown that the F-specific monoclonal antibody hu1F5 provides protection in nonhuman primate models, with efficacy comparable to that of m102.4. This supports the potential for complementary or combination targeting of both F and RBP glycoproteins [117]. hu5B3.1, a humanized antibody derived from a murine precursor targeting the F protein, provides survival benefits in ferrets following high-dose oral-nasal challenges with NiV or HeV [118]. Recent hu1F5 and hu12B2, humanized monoclonal antibodies, along with NiV41/41‑6, human neutralizing antibodies targeting the receptor-binding protein (RBP), demonstrate protective effects in hamsters and AGMs [18]. Combinations of fully human RBP-specific mAbs, such as HENV-26/32 and HENV-103/117, provide synergistic protection and enhance coverage against NiV and HeV in ferrets and hamsters [118]. In India, m102.4 has been integrated into standard operating procedures for high-risk exposures. Kerala has commenced collecting convalescent sera from NiV survivors to aid the development of indigenous monoclonal antibodies specifically designed for locally circulating clade I strains, in collaboration with the ICMR and public sector partners [119,120]. Global supply limitations and restricted administration windows are significant constraints, necessitating prioritizing scale-up and regionalized mAbs development pipelines. Additional challenges include high manufacturing costs, potential immunogenicity with repeated dosing, and limited CNS penetration, which may restrict efficacy in neuroinvasive disease [121]. This is particularly significant considering the neurotropic and brainstem-invasive characteristics of NiV infection [60,82]. Taken together, while the development of indigenous monoclonal antibodies may enhance regional availability and logistics, it does not inherently address the biological and pharmacoeconomic limitations.

**Table 6:** Vaccine and therapeutics.

Intervention	Modality	Target/Mechanism	Model	Outcome	Stage	References
***Vaccines***						
HeV-sG (Equivac HeV)	Soluble G glycoprotein subunit vaccine	Soluble HeV G	Ferrets, cats, AGM	Highly protected (NiV-B/M)	Licensed (veterinary)	[122]
ChAdOx1 NiV-B	ChAdOx1 adenoviral vector	NiV-B G	Syrian hamsters	Sterilizing immunity	Phase 2 (Bangladesh, 2025)	[123]
rVSVΔG-NiV-G/F	Recombinant VSV vector	NiV G and F	AGMs	Highly protected (NiV-B)	Preclinical	[126]
rVSV-EBOV-GP-NiV-G	Recombinant VSV vector	NiV G + EBOV GP	AGMs	Protection (NiV-M)	Preclinical	[124]
rMV-NiV-G	Recombinant measles virus vector	NiV G	AGMs	Neutralizing Ab	Preclinical	[128]
rRABV-NiV-G	Recombinant rabies virus vector	NiV G	Mice	Seroconversion; dual vaccine	Preclinical	[129]
BoHV-4-NiV-G/F	BoHV-4 herpesvirus vector	NiV G or F	Pigs	Protection; livestock strategy	Preclinical	[130]
NiV-VLP	Virus-like particle	G, F, M	Syrian hamsters	Highly protected	Preclinical	[131]
mRNA-sHeV-G	mRNA vaccine	Soluble HeV G	Syrian hamsters	Partial protection	Preclinical	[125]
mRNA-1215	mRNA vaccine	NiV G and F	—	Under development	Early stage	[139]
Ferritin NP	Ferritin nanoparticle vaccine	NiV G (NiV-M+ NiV-B)	—	Multivalent cross-strain	Preclinical	[137]
***Antiviral drugs***						
Remdesivir	Nucleoside analogue	RdRp inhibition	AGM; Kerala 2023	Highly protected	Compassionate use	[111]
Favipiravir	Nucleoside analogue	RdRp inhibition	Hamster NiV-M	Highly protected	Preclinical	[112]
Ribavirin	Guanosine analogue	RNA replication inhibition	Malaysia 1998–1999	Decreased mortality	Empirical	[148]
Balapiravir	Cytidine analogue	RdRp inhibition	—	In vitro activity only	Preclinical	[39]
Poly(I:C)12U	IFN inducer	IFN-α/β stimulation	Hamster	Increased survival	Preclinical	[114]
***Monoclonal antibodies***						
m102.4	Fully human mAb (phage-display)	NiV/HeV G (RBD)	AGM, ferret	Highly protected	Phase 1 complete	[115,116]
hu5B3.1	Humanized mAb (murine 5B3)	NiV/HeV F	Ferret NiV-M	Highly protected	Preclinical	[118]
hu1F5	Humanized mAb (murine 1F5)	NiV/HeV F	AGM NiV-B	Highly protected	Preclinical	[117]
NiV41/41-6	Fully human mAb (donor-derived)	NiV/HeV RBP (neutralization)	Hamster	Highly protected	Preclinical	[149]
HENV-26/32	Fully human mAb (donor-derived)	HeV G (NiV-G cross-reactive)	Ferret	Post-exposure protection	Preclinical	[118]
HENV mAb cocktail (HENV-103 and HENV-117)	mAb combination	HeV RBP (NiV cross-reactive)	Hamster	Synergistic protection	Preclinical	[150]
hu1F5	Humanized mAb (murine 1F5)	NiV F	AGM, hamster	Highly protected	Preclinical	[117]
hu12B2	Humanized mAb (murine 1F5)	NiV F	Hamster	Highly protected	Preclinical	[117]
F mAb cocktail (hu1F5 + hu12B2)	mAb combination	NiV F	Hamster	Partial protection (80% survival)	Preclinical	[117]
***Combination therapies & preparedness***						
Ribavirin + chloroquine	Drug combination	RdRp + entry	Hamster	No protection	Preclinical	[148]
Remdesivir + ICU care	Antiviral + support	Kerala 2023	Humans (early cases)	Operational	Compassionate use	[151]
ChAdOx1 stockpile	Vaccine preparedness	Prevention	Bangladesh/India	100k doses reserved	Ongoing	[136]
Bat surveillance	Surveillance	Spillover prediction	Kerala/West Bengal	Early-warning	Ongoing	[40,152]
HCW training	IPC programme	Containment	Kerala	Zero HCW infections	Ongoing	[17]

Vaccine development primarily focuses on platforms that target the NiV G and F glycoproteins. A soluble HeV G subunit (HeV‑sG) vaccine offers significant cross‑protection against both NiV and HeV in cats, ferrets, swine, and AGMs, serving as the foundation for Equivac HeV, the sole licensed henipavirus vaccine for veterinary application in horses in Australia [39,122]. However, vector-based platforms currently dominate in the domain. ChAdOx1 NiV-B elicited substantial neutralizing antibodies and conferred total protection in hamsters against both NiV-B and NiV-M [123]. Additionally, various recombinant vesicular stomatitis virus (rVSV) constructs, including rVSV-ΔG-NiV-BF/GFP and chimeric replication-competent vectors, rVSV-EBOV-GP-NiV-G have shown efficacy in protecting AGMs from lethal NiV challenges, including NiV-B, while resulting in minimal clinical disease [124–126]. In contrast, rVSV constructs that express both NiV G and F glycoproteins have demonstrated neurovirulence in murine models, making them less suitable as vaccine candidates [127]. Recombinant vectors, including measles virus expressing NiV‑G (rMV‑NiV‑G), rabies virus encoding NiV‑G (rRABV‑NiV‑G), and bovine herpesvirus 4 expressing NiV‑G or NiV‑F (BoHV‑4‑NiV‑G/F), provide protective effects in AGMs, mice, and pigs, respectively, and present strategies focused on dual diseases or livestock [128–130]. Vaccines based on virus-like particles (VLPs) containing NiV G, F, and M induce robust neutralizing antibody levels and confer complete protection in Syrian hamsters [131]. Several in silico studies have identified peptide- and epitope-based constructs targeting N, V, F, G, and M epitopes to elicit B- and T-cell responses; however, they remain in the preclinical stage [106,132–135]. mRNA platforms, such as the HeV‑sG mRNA vaccine and the mRNA‑1215 NiV G/F candidate, demonstrate partial protection in hamsters. CEPI and the WHO R&D Blueprint prioritize these technologies for outbreak-responsive stockpiles [136]. Recent ferritin-based nanoparticle vaccines exhibiting NiV G antigens from both NiV-M and NiV-B strains enhance the potential for broadly protective, multivalent henipavirus vaccines [137].

India may play a crucial translational role in this context. The Serum Institute of India (SII) leverages its COVID-19-era ChAdOx platform expertise to produce ChAdOx1 NipahB in collaboration with the University of Oxford and CEPI [136]. This initiative supports the inaugural phase 2 NiV vaccine trial in Bangladesh and aims to create a reserve of ~100,000 doses in Pune for swift distribution in South Asia [136]. National guidelines from the National Centre for Disease Control (NCDC) and the Kerala State Medical Board recommend the early use of remdesivir and favipiravir on compassionate grounds, in conjunction with ribavirin for severe cases. The 2023 Kerala outbreak demonstrated improved outcomes, likely due to several factors, including timely access to antivirals, intensive supportive care, and enhanced clinical management; however, the precise role of antiviral therapy remains unclear [110]. ICMR and regional institutes are simultaneously developing India-specific mAbs against clade I NiV utilizing survivor samples, while also investigating local manufacturing partnerships to decrease reliance on imported biologics [119,120,138]. However, major translational barriers persist, particularly in India. Despite recent advancements, all NiV vaccines are still unlicensed for human use, and most therapeutics are limited to preclinical or early-phase trials. Conducting large-scale vaccine efficacy trials is inherently challenging due to the sporadic, geographically focal, and unpredictable nature of NiV outbreaks. The lack of a sustained commercial market for vaccines aimed at an intermittently emerging pathogen limits late-stage development and scaling efforts. Manufacturing constraints and the need to ensure broad protective efficacy against circulating and potentially emerging NiV strains in the absence of adequate clinical efficacy data continue to pose challenges for vaccine development and evaluation. This highlights the need for adaptive trial designs, surrogate immune correlates, emergency-use pathways, and globally coordinated stockpiling frameworks that incorporate Indian manufacturing and outbreak experience [106,139].

## 10. Prevention, control, and one health strategies

**Fig 6:**
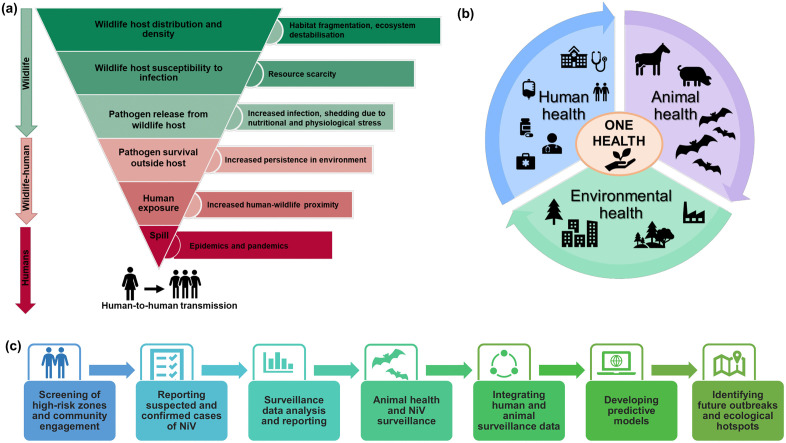
One Health framework and strategies for pandemic preparedness. **(a)** The schematic depicts the process of zoonotic spillover from wildlife reservoirs, specifically fruit bats, to humans. The phenomenon is influenced by ecological disruption, habitat encroachment, agricultural intensification, and heightened human-animal interactions. **(b)** The One Health approach integrates surveillance of human, animal, and environmental factors to mitigate the risk of zoonotic spillover. **(c)** The figure presents a systematic integrated framework for the early detection and prevention of NiV outbreaks. The figure was created by the authors. The figure design, layout, and annotations were created by the authors.

Due to the lack of licensed antivirals or vaccines, prevention is fundamental in controlling NiV transmission. The epidemiology of India, characterized by peri-domestic exposure, nosocomial transmission, and seasonal spillovers, requires a comprehensive One Health approach that integrates human, animal, and environmental health systems (Fig 6). The recurrent NiV outbreaks in Kerala from 2018 to 2023 serve as a significant model for global outbreak containment strategies. In 2018, the state experienced an outbreak with 18 confirmed and four probable cases, an 89% CFR, and over 2600 contacts monitored for 21 days. The outbreak was declared over within weeks, with an estimated reproduction number of 0.4 [99,138]. Following a single-case in 2019, one in 2021, and six cases with two deaths in 2023, standard protocols were implemented for rapid RT-PCR and ELISA testing, ring containment, risk-stratified quarantine for 21 days, and intensive contact tracing, with 1260–1288 contacts under surveillance in 2023 alone [138,140]. High-concentration sodium hypochlorite and ethanol solutions were used to fully inactivate NiV within minutes [40,100]. Kerala has incorporated this evidence into NCDC-aligned IPC guidance, resulting in a significant reduction in case fatalities between the 2018 and 2023 outbreaks [17,110,138]. The IDSP facilitated decentralized surveillance, while real-time coordination with the NIV, Pune, and the activation of district rapid response teams contributed to the closure of outbreaks within a single incubation period [17]. Stringent infection prevention and control measures, such as the establishment of designated NiV intensive care units, the use of powered air-purifying respirators, and the implementation of chemical disinfection protocols, were essential for mitigating nosocomial amplification. Primary prevention has concentrated on minimizing interactions between bats and humans. In India, risk communication has focused on avoiding consumption of bat-contaminated fruits, promoting safe food-handling practices, and discouraging fear-driven ecological responses, such as the destruction of roosts, which could inadvertently heighten spillover risk. The annual bat surveillance carried out by ICMR-NIV in Kerala and West Bengal, including qPCR and serology, has enhanced early-warning capabilities, supported by ecological risk mapping of roosts near human settlements. India’s response has been characterized by effective intersectoral coordination. Health departments, animal husbandry services, forest authorities, and local governments have collaborated to implement surveillance, quarantine measures, and public awareness initiatives. Kerala established multisectoral committees for clinical management, laboratory coordination, risk communication, and digital surveillance during outbreaks, demonstrating institutional learning from COVID-19 and previous NiV incidents. Effective management of NiV necessitates a shift from reactive containment to proactive strategies. Institutionalization of climate-informed surveillance, bat ecology monitoring, land-use governance, and community engagement is essential to address upstream drivers, including deforestation, urban expansion, and climate variability. Enhancing laboratory capacity (BSL-3/4), broadening syndromic surveillance in under-resourced areas like parts of West Bengal, and integrating One Health principles into national preparedness strategies are crucial to avert the increasing risk of NiV spillovers. India’s experience illustrates that NiV prevention encompasses not only biomedical factors but also socio-ecological dimensions. India is a key partner in CEPI and WHO initiatives to develop and stockpile NiV countermeasures, such as Nipah mAbs and ChAdOx1 NipahB vaccine reserves, for rapid deployment during future outbreaks [136]. The success of Kerala demonstrates that sustained investment in surveillance, preparedness, and intersectoral coordination can effectively manage high-fatality zoonoses, providing essential insights for preventing future pandemics like NiV. Key knowledge gaps and priority research directions are summarized in Box 1.

**Box 1. Key knowledge gaps and priority research directions in Nipah virus.**

1. Epidemiology and outbreak dynamics
  - The true burden of subclinical/silent infections beyond known hotspots remains unquantified.
  - Inadequate genomic surveillance constrains tracking of regional NiV evolution.
  - Limited resolution of intra-country heterogeneity and spillover hotspots.
  - Need for standardized, real-time, integrated surveillance systems.
2. Reservoir ecology and emerging drivers
  - Limited longitudinal data on viral shedding dynamics of *Pteropus medius*.
  - Climate and land-use drivers of spillover remain poorly quantified.
  - Bat-human interface in peri-domestic and agricultural settings remains poorly defined.
3. Transmission and spillover pathways
  - Determinants of variable human-to-human transmission remain unclear.
  - Relative contribution of zoonotic vs secondary transmission is poorly quantified.
  - Limited integration of ecological, behavioral, and climatic drivers.
4. Pathogenesis and clinical outcomes
  - Mechanisms underlying respiratory vs neurological tropism remain unclear.
  - Host immune correlates of survival and long-term sequelae remain undefined.
  - Mechanisms of vascular leakage and ARDS in high CFR settings are unknown.
  - Lack of validated prognostic biomarkers.
  - Limited translational models that accurately reflect human disease.
5. Diagnostics, therapeutics, and vaccines
  - Absence of rapid, field-validated diagnostic tools for outbreak scenarios.
  - No licensed vaccines or antivirals with proven human efficacy against circulating strains.
  - Constraints in clinical trials due to sporadic outbreaks.
6. Priority research and One Health strategies
  - Establish integrated One Health genomic surveillance.
  - Conduct longitudinal ecological monitoring at high-risk interfaces.
  - Develop human-relevant models, such as iPSC-derived organoids.
  - Advance trial-ready vaccines/therapeutics employing scalable deployment frameworks.
  - Strengthen integration of national and state-level surveillance and response systems.

## 11. Conclusion

NiV is a major emerging pathogen in India, characterized by repeated zoonotic spillovers, elevated case-fatality ratios, and established human-to-human transmission, indicating continuous epidemic potential. India’s epidemiology, characterized by recurrent direct bat-to-human spillover and nosocomial amplification without a stable intermediate host, fundamentally contrasts with the pig-amplified outbreaks in Malaysia and the sap-associated transmission in Bangladesh. This highlights the necessity for context-specific control strategies. Climatic variability, land-use changes, and increasing interactions between bats and humans heighten the likelihood of future spillover events. The consistent success of Kerala in managing outbreaks underscores the efficacy of integrated One Health strategies that incorporate bat-human surveillance, climate-informed risk modeling, decentralized diagnostics, infection prevention and control, and community involvement. Nonetheless, preparedness varies significantly among regions. Continuous investment in One Health-integrated surveillance, field-ready diagnostics, and vaccines, antivirals, and monoclonal antibodies, in alignment with WHO and CEPI initiatives, is crucial for transitioning from reactive containment to a sustained approach to resilience against future zoonotic outbreaks.

**Key Learning Points**

- Nipah virus (NiV) causes recurrent high-fatality zoonotic outbreaks in India.
- Genomic and epidemiological studies reveal the NiV-Bangladesh clade as the dominant lineage.
- NiV spillovers peak post-monsoon via fruit proximity in Kerala and winter sap events in West Bengal.
- Climate change and habitat destruction intensify bat-human interfaces.
- One Health approach is key for surveillance and outbreak response.

**Top Five Papers**

- Chua KB, Bellini WJ, Rota PA, Harcourt BH, Tamin A, Lam SK, et al. Nipah virus: a recently emergent deadly paramyxovirus. Science (New York, NY). 2000;288(5470):1432–5. Epub 2000/05/29. https://doi.org/10.1126/science.288.5470.1432. PubMed PMID: 10827955.
- Chadha MS, Comer JA, Lowe L, Rota PA, Rollin PE, Bellini WJ, et al. Nipah virus-associated encephalitis outbreak, Siliguri, India. Emerging infectious diseases. 2006;12(2):235–40. Epub 2006/02/24. https://doi.org/10.3201/eid1202.051247. PubMed PMID: 16494748; PubMed Central PMCID: PMCPMC3373078.
- Sahay RR, Patil DY, Chenayil S, Shete AM, Ps KS, Mohandas S, et al. Encephalitis-predominant Nipah virus outbreaks in Kerala, India during 2024. J Infect Public Health. 2025;18(7):102782. Epub 2025/04/21. https://doi.org/10.1016/j.jiph.2025.102782. PubMed PMID: 40253778.
- Hassan MZ, Rojek A, Olliaro P, Horby P. Improving clinical care of patients in Nipah outbreaks: moving beyond ‘compassionate use’. The Lancet regional health Southeast Asia. 2025;33:100527. Epub 2025/01/27. https://doi.org/10.1016/j.lansea.2024.100527. PubMed PMID: 39866590; PubMed Central PMCID: PMCPMC11755010.
- Epstein JH, Anthony SJ, Islam A, Kilpatrick AM, Ali Khan S, Balkey MD, et al. Nipah virus dynamics in bats and implications for spillover to humans. Proc Natl Acad Sci U S A. 2020;117(46):29190–201. Epub 2020/11/04. https://doi.org/10.1073/pnas.2000429117. PubMed PMID: 33139552; PubMed Central PMCID: PMCPMC7682340.
